# Microwave and blanch‐assisted drying of white yam (*Dioscorea rotundata*)

**DOI:** 10.1002/fsn3.249

**Published:** 2015-06-10

**Authors:** Ernest Ekow Abano, Robert Sarpong Amoah

**Affiliations:** ^1^ Department of Agricultural EngineeringUniversity of Cape CoastCape CoastGhana

**Keywords:** Ascorbic acid, blanching, drying models, microwave, moisture diffusivity, nonenzymatic browning, White yam

## Abstract

The effect of microwave and blanch pretreatments on the drying kinetics and quality of white yam (*Dioscorea rotundata*) was investigated. Yam cubes destined for hot air drying at temperatures 70–90°C were predried in a domestic microwave or blanched in hot water for 1–5 min. Microwave pretreatment time had a positive significant effect on drying rate but both pretreatments had a negative influence on the ascorbic acid content and the nonenzymatic browning. The optimum drying conditions were a microwave pretreatment time of 5 min and a temperature of 70°C and a blanching time of 1 min at a temperature of 80°C. Among the models fitted, the Midilli et al. and the Page models gave the best fits for yam cubes predried with microwave and blanch, respectively. The effective moisture diffusivity for microwave‐assisted drying increased from 1.05 × 10^−8 ^m^2 ^s^−1^ to 2.00 × 10^−8^ m^2 ^s^−1^ while the hot water blanched samples decreased from 1.53 × 10^−8^ to 8.81 × 10^−9 ^m^2 ^s^−1^ with time. The study demonstrates that microwave‐assisted drying could be used to enhance heat and mass transfer processes to produce better quality dried yam products.

## Introduction

White yam (*Dioscorea rotundata*) is an important food security crop in West Africa being a nutritious, economical, and a healthy crop. It is used widely in Chinese medicine due to its anticarcinogenic and antithrombotic functionality from steroidal saponins (Liu et al. 1995; Hu et al. 2007; Yang and Lin 2008; Xiao et al. 2012). FAO statistics show that Ghana is the second highest producer of yam in the world after Nigeria and the crop contributes about 16% of the country's Agricultural Gross Domestic Product (GDP) (FAOSTAT, 2012). Yam is one of the most important dietary sources of energy composed mainly of starch, with some proteins, lipids, vitamin C, essential minerals, antiaging, and fertility promotion properties (Lasztity et al. 1998). In the diet of most Ghanaians, yam provides the third most important source of energy, accounting for 20% of total caloric intake (FAOSTAT 2012).

Yams are highly perishable because of its relatively high moisture content (50–80%), high respiration rates (Noamesi 2008), and the absence of no protective cuticle. It is estimated that postharvest losses of yam are more than 20% and are mainly due to rot caused by fungus during storage (MOFA, 2007). Postharvest losses significantly affect farmers' and traders’ income and threaten food security interventions. Hence, harvested yams must be consumed within a few weeks or be processed adequately into flour by peeling, slicing, blanching, and drying. Drying is one of the technologies, which can be used to produce quality products and to reduce postharvest losses associated with yam storage. Microwave and blanching have been widely used in food processing technologies. Several researches have provided strong evidence that microwave‐assisted drying is ideal for fruits and vegetables (Prothon et al. 2001; Schiffmann 2001; Zhang et al. 2006; Vadivambal and Jayas 2007; Contreras et al. 2008; Figiel 2009; Abano et al. 2013). The objective of microwave drying is to speed up drying process, increase mass transfer, and produce good quality products while blanching prior to processing fruits and vegetables accelerate drying rate*,* prevents colour changes, softens the texture, denatures enzymes, and destroys contaminating microorganisms (Jayaraman and Gupta 2006; Therdthai and Zhou 2009). Lu et al. (1998) studied microwave drying of *Dioscorea alata* tubers and its associated moistures distribution. Lin et al. (2007) investigated the far‐infrared‐radiation‐assisted freeze drying, while Falade et al. (2007) studied hot air drying of *Dioscorea alata and Dioscorea rotundata* slices. Xiao et al. (2012) similarly investigated the superheated steam blanching of hot air drying of *Dioscorea alata* slices. Hot air drying kinetics of *Dioscorea alata* varieties has been investigated by Torres et al. (2012). However, studies on microwave and blanch‐assisted drying kinetics and quality of white yam (*Dioscorea rotundata*) are limited if not unavailable. In industrial food processing, the goal is to satisfy the consumer demand in terms of quality at a minimum cost. It is usually characterized by its nutritional value in terms of vitamin loss and protein denaturation, safety (browning, level of microbiological and toxicological contaminants), and acceptability with respect to colour, aroma, texture etc. (Timoumia et al. 2007; Vadivambal and Jayas 2007).

Therefore, the objective of the present study was to investigate the effect of microwave and blanch time and drying temperature on hot air drying kinetics and quality attributes such as ascorbic acid and nonenzymatic browning of dried white yam slices.

## Materials and Methods

### Sample preparation

White yams (*Dioscorea rotundata)* were obtained from the University of Cape Coast Science market. Prior to the test, the yams were peeled and cut into cubes of size 3 cm using stainless steel knife. The sliced samples were stored in a refrigerator at a temperature 4°C in order to slow down the physiological and chemical changes. The initial moisture content was obtained from drying 30 g samples in an oven at 105°C for 24 h (AOAC, 1990). The drying experiment were completed within 2 weeks and kept in a freezer for further analysis.

### Experimental design

A 2‐ factor, 3‐level factorial design was used for both the microwave‐assisted and the blanched drying experiments. Samples were either pretreated with a microwave or blanched at 1, 3, and 5 min after which it was subjected to drying at temperatures of 70, 80, and 90°C. The effect two factors: temperature (*X*_1_) and pretreatment time (*X*_2_) was investigated in this study.

### Blanching and microwave pretreatments

Ninety grams of yam cubes were placed in a stainless steel sieve bowl and exposed to the steam of boiling water at 100°C for the various times (1–5 min). After blanching, the surface moisture was blotted with absorbent paper and allowed to cool, after which it was subjected to hot air drying. For the microwave pretreatment, a domestic microwave with a maximum power output of 800W at 2450 MHz was used to predry the same amount of yam cubes before hot air drying as described below. Each experiment was in triplicate.

### Drying procedure

The pretreated yam cubes were transferred to a hot air cabinet dryer set at the various temperatures at air velocity of 0.5 m/s. These temperatures were chosen based on the preliminary experiments carried out. The dryer was run idle for an hour prior to the drying experiment to achieve steady state conditions. The masses of the drying samples were monitored every 30 min at the initial stages and later at 1 h intervals of drying until constant mass was observed using a digital balance with an accuracy of ±0.001 g.

### Determination of ascorbic acid

The ascorbic acid content in the fresh and dried samples were determined volumetrically by the redox titration method using potassium iodate according the protocol of Brody (1994).

### Nonenzymatic browning determination

A modified method of (Cernîsev 2010) was used to determine the browning index (BI) of the dried tubers. The extent of browning was evaluated as browning index measured as absorbance at 440 nm. Brown pigments were extracted from the test portions of the dried yam samples. A two‐gram sample was grounded into fine powder, after which 50 mL of ethanol (60%, v/v) was added and allowed to stand for 12 h. After 12 h, the mixture was stirred slowly and then filtered through 0.45 *μ*m nylon filter membrane. Browning index of the filtrate was estimated with a spectrophotometer against 60% ethanol as blank. All samples were extracted in triplicate.

### Drying kinetics expressed in terms of empirical models

The drying kinetics of yam cubes were expressed in terms of empirical models, where the experimental data obtained were plotted in the form of a dimensionless moisture ratio (MR) against drying time (expressed in minutes). The MR of the yam cubes was determined using the following equation:

(1) $$\text{MR}\;=(\frac{M-M_{e}}{M_{o}-M_{e}})$$

where MR is the moisture ratio, *M* is the moisture content at a specific time (*t*) (g water/g dry matter), *M*_*o*_ is the initial moisture content (g water/g dry matter), *M*_*e*_ is the equilibrium moisture content (g water/g dry matter).

Three empirical drying models widely used in scientific literature: Page, Henderson and Pabis, and Midilli et al. were fitted to the experimental data set (MR, *t*) shown in Table 1 to describe the drying kinetics of yam cubes. Three primary criteria were used to determine the goodness of fit to the models; the correlation coefficient (*R* ^2^), the root mean square error (RMSE), and the reduced chi‐square (*χ* ^2^). The highest *R* ^2^, lowest *χ* ^2^, and RMSE were adjudged the best fit (Karaaslan and Tuncer 2008)

**Table 1:** Thin layer drying models applied

Model name	Model expression	References
Page	MR = exp(−*kt* ^*n*^)	Diamente and Munro (1993)
Henderson & Pabis	MR = *a* exp(−*kt*)	Ghodake et al. (2006)
Midilli et al.	MR = *a* exp(−*kt* ^*n*^) + *bt*	Midilli et al. (2002)

(2) $$R^{2}=1-\left[\frac{\sum_{i\;=1}^{N}{\left({\text{MR}}_{\mathrm{pre},i}-{\text{MR}}_{\exp,i}\right)}^{2}}{\sum_{i\;=1}^{N}{\left({\text{MR}}_{\mathrm{pre},i}-{\text{MR}}_{\mathrm{pre},i}\right)}^{2}}\right]$$

(3) $$X^{2}=\frac{\sum_{i\;=1}^{N}{\left({\text{MR}}_{\exp,i}-{\text{MR}}_{\mathrm{pre},i}\right)}^{2}}{N-z}$$

(4) $$\text{RMSE}\sqrt{\frac{1}{N}\sum_{i\;=1}^{N}{\left({\text{MR}}_{\mathrm{expt},i}-{\text{MR}}_{\mathrm{pred},i}\right)}^{2}}$$

where MR_exp,*i*_ and MR_pred,*i*_ are the experimental and predicted dimensionless MR respectively, *N* is the number of observations, and *z* is the number of model constants. The drying rate constants, *k*, and coefficients of the model equations (*a*,* n*,* b*) were determined with nonlinear regression of SPSS 16.0 (SPSS, 2007) and the goodness of fit of the curves were determined with correlation analysis.

### Determination of moisture diffusivity

Fick's second equation of diffusion was used to calculate the effective moisture diffusivity, considering a constant moisture diffusivity, infinite slab geometry, and uniform initial moisture distribution (Crank 1975):

(5) $$\text{MR}=\frac{8}{\pi^{2}}\sum_{n\;=\;0}^{\infty }\frac{1}{\left(2n+1\right)}\text{exp}\left(-\frac{\left(2n+1\right)\pi^{2}}{4L^{2}}D_{\mathrm{eff}}t\right)$$

where *D*_eff_ is the effective diffusivity (m^2^/s), and *L* is half the thickness of slice of the sample (m). The equation (5) can be simplified to equation (6):

(6) $$\text{MR}=\frac{8}{\pi^{2}}\text{exp}\left(-\frac{\pi^{2}D_{\mathrm{eff}}t}{4L^{2}}\right)$$

The *D*_eff_ of the yam cubes was obtained from the slope (*K*) of the graph of InMR against the drying time. InMR versus drying time results in a straight line with a negative slope and *K* is related to *D*_eff_ by (eq. (7)).

(7) $$K\;=\frac{\pi^{2}D_{\mathrm{eff}}t}{4L^{2}}$$

### Optimization of the drying process

The optimization of the drying process was performed using a multivariate response method called overall desirability index, DI (Meyers and Montgomery 2002) using equation (8). The DI represents the desirability index for each response variable (*Y*_*i*_) and it is a multicriteria optimization approach used to show how desirable the various responses are.

(8) $$\text{DI}={\left[\prod_{i\;=1}^{3}di\left(Y_{i}\right)\right]}^{1\;/\;3}$$

The DI ranges between 0 and 1 with 0 being the least desirable, while 1 is the most desired. Maximization of DI value is the goal in optimization studies. The optimization process incorporates goals and priorities for the independent and response variables. For this study, the goal for the independent variables was at any level within the range of the design values. However, in the case of the response variables, the goal was minimization of drying time and browning index, and maximization of ascorbic acid content.

### Statistical analysis

A model was fitted to the mean values of the experimental results to get the regression equations with Design Expert (2010). The statistical significance of the model terms was checked out at a probability of 0.05. The accuracy of the model to describe the response variables was diagnosed against the normal probability plots of the residuals, the predicted versus actual plots, and the coefficients of determination (*R* ^2^) values. The 3‐D plots for the factors were generated for the various responses.

## Results and Discussion

### Effect of microwave and blanching pretreatments on drying kinetics of yam cubes

**Figure 1:**
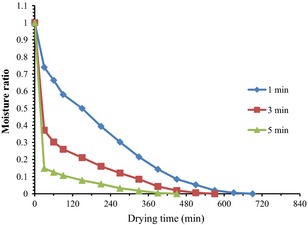
Variation in moisture ratio versus drying time for the various microwave pretreatment times at 70°C.

The effect of microwave pretreatment time on drying time for the 70°C dried samples is shown in Figure 1. The initial average moisture content of the white yam (*Dioscorea rotundata*) was found to be 1.50 g moisture/g dry matter (d.b), which decreased to 0.12 g moisture/g dry matter (d.b) after dehydration. The drying followed a falling rate regime and the increase in microwave pretreatment time accelerated the drying process and increased energy efficiency. As microwave pretreatment time increased, moisture removal also increased and ultimately resulted in the reduction in drying time (Table 2). Drying time reduced as the microwave pretreatment time increased from 1 to 5 min for the various temperatures used in this study. This means that there was significant savings in time as microwave pretreatment time increased.

**Table 2:** Drying time (DT), Browning index (BI), and Absorbic acid content (AA) (mg/1010 g) of yam pretreated with microwave and blanching

*T* (°C)	PT (min)	Microwave			Blanching		
		DT (min)	BI (Abs unit)	AA (mg/100 g)	DT (min)	BI (Abs unit)	AA (mg/100 g)
70	5	390	0.048	1.3728	720	0.029	0.8448
70	1	630	0.051	1.9008	660	0.053	1.4784
70	3	510	0.042	1.4784	600	0.044	1.3728
80	3	450	0.035	1.1616	600	0.065	1.2672
90	1	450	0.089	1.0560	540	0.127	1.3728
90	3	390	0.063	1.1616	540	0.080	0.8448
70	1	630	0.048	1.6896	600	0.052	1.3728
80	1	510	0.070	1.2672	600	0.066	1.3728
90	1	450	0.073	1.2672	480	0.098	1.0560
70	5	420	0.067	1.2672	720	0.031	0.8448
90	5	330	0.086	1.0560	600	0.066	0.5300
80	5	330	0.080	1.1616	720	0.050	1.0560
90	5	330	0.112	0.8448	600	0.066	0.5280
80	1	570	0.029	1.4784	600	0.074	1.2672
80	3	450	0.109	1.2672	600	0.064	1.3728
70	3	510	0.072	1.3728	720	0.046	1.2672
90	3	390	0.097	1.2672	540	0.084	1.2672

Temperature (T) in °C; Pretreatment time (Pt) in min; Drying time (DT) in min; Nonenzymatic browning index (BI) in Absorbance units and Ascorbic acid content (AA) in mg/100 g.

The results agree with what was reported by Rayaguru and Routray (2011), Bai‐Ngew et al. (2011) and Karaaslan and Tuncer (2008) for microwave drying of pandanus amaryllifolius leaves, durian chips, and spinach, respectively. Formation of porous structure in the tissues of yam as a result of electromagnetic waves application has been noted to be the plausible reason for the accelerated drying with microwave.

**Figure 2:**
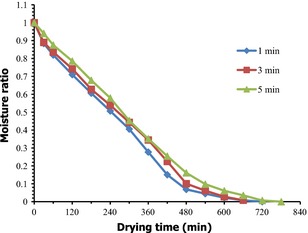
Variation in moisture ratio versus drying time for the various blanching times at 70°C.

On the contrary, blanching decreased the rate of moisture removal thereby leading to increase in drying time (Fig. 2). The increase in moisture content during blanching has been reported for yam and *Dioscorea schimperiana* (Quansah et al. 2010; Leng et al. 2011). When the yam was blanched the starch may have gelatinized at the higher temperature. Higher degree of starch gelatinization affects tissue structure and increases the internal resistance to moisture movement (Mate et al. 1998).

### Effect of microwave and blanching pretreatments on moisture diffusivity

**Figure 3:**
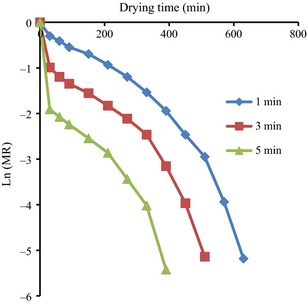
Variation in Ln(MR) against drying time plot for the various microwave pretreatment times at temperature of 70°C.

The variation in Ln(MR) against drying time plot for the various microwave pretreatment time is shown in Figure 3. The slopes of the straight line generated by the plot of Ln(MR) against drying time were used to determine the effective moisture diffusion values. The effective moisture diffusivity coefficient, D_eff_, increased with pretreatment time as with corresponding values of 1.05 × 10^−8 ^m^2 ^s^−1,^ 1.22 × 10^−8 ^m^2 ^s^−1^ and 1.57 × 10^−8 ^m^2 ^s^−1^ at 1, 3, and 5 min microwave pretreatment times for samples dried at 70°C. A similar increase with increasing hot air temperature was observed for the diffusivity values of the various air temperature ranges (Table 3). The values of the *D*_eff_ obtained from this research lie within the general range of 10^−12^–10^−8 ^m^2 ^s^−1^ for drying of food materials (Doymaz 2010).

**Table 3:** Effect of temperature and pretreatment time on effective moisture diffusivity and their coefficient of determination

Temper‐ature (°C)	Pretreatment time (min)	*D*_eff_ (m^2 ^s^−1^)		*R* ^2^	
		Microwave	Blanching	Microwave	Blanching
70	1	1.05 × 10^−8^	1.02 × 10^−8^	0.9136	0.8139
	3	1.22 × 10^−8^	1.02 × 10^−8^	0.9263	0.9123
	5	1.57 × 10^−8^	8.81 × 10^−9^	0.8809	0.8790
80	1	1.26 × 10^−8^	1.15 × 10^−8^	0.8939	0.9219
	3	1.60 × 10^−8^	1.08 × 10^−8^	0.9476	0.9173
	5	1.61 × 10^−8^	1.00 × 10^−8^	0.9109	0.9133
90	1	1.69 × 10^−8^	1.53 × 10^−8^	0.9296	0.9045
	3	1.70 × 10^−8^	1.22 × 10^−8^	0.9767	0.9356
	5	2.00 × 10^−8^	1.06 × 10^−8^	0.9363	0.9342

**Figure 4:**
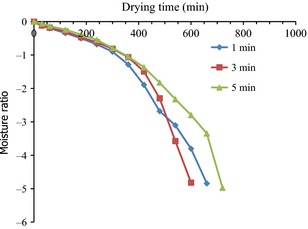
Variation in Ln(MR) against drying time plot for the various blanching time at temperature of 70°C.

The variation in Ln(MR) against drying time plot for the various blanching time is also shown in Figure 4. The effective moisture diffusivity coefficient, *D*_eff_ decreased with increase in blanching time (Fig. 4). A microstructure analysis with scanning electron microscope (SEM) of hot water and superheated steam blanched sweet potatoes revealed no pores, but formation of starch granules on the surface of the test samples (Hong‐Wei et al. 2009). This shows that the longer the samples were blanched, the higher the formation of homogenous compact structure to impede heat and mass transfer, leading to lower moisture diffusivity rates.

### Modeling of the drying curves

The dimensionless moisture ratio against drying time for the experimental data at various pretreatment times and air temperatures was fitted to the Page, Henderson and Pabis, and Milli et al. models available in the literature. The results of such a fitting of the experimental data for the microwave pretreated samples dried at 70°C are displayed in Table 4, which show the values of the estimated constants with their corresponding statistical *R* ^2^, *χ* ^2^, and RMSE values characterizing each fitting. From the results obtained, it is evident that the experimental data fitted to the models used in this study. The correlation coefficients obtained are in the range of 0.878–1.000. This means that the three models could satisfactorily describe the microwave‐assisted hot‐air drying of yam cubes. The relatively high values of correlation coefficients, low reduced chi‐square, and low root mean square errors indicate a good predicting capacity for the temperature tested over the entire duration of the drying process. Among the models examined, the Milli et al. model was observed to be the most appropriate one for all the experimental data with the highest value for the coefficient of determination (*R* ^2^) and the lowest reduced chi‐square (*χ* ^2^) and RMSE. The estimated parameters and statistical analysis of the models examined for the different drying conditions are illustrated in Table 4. It was observed that the value of drying rate constant (*k*) increased with microwave pretreatment time. This implies that drying rate potential increased with increase in microwave pretreatment time.

**Table 4:** Parameters and statistical results for the various drying models for microwave‐pretreated samples dried at 70°C

Model name	*P*_t_	Model constants	*R* ^2^	RMSE	*χ* ^2^
Page	1	*k*: 0.010, *n*: 0.883	0.980	0.0439	0.0022
	3	*k*: 0.203, *n*: 0.434	0.987	0.0302	0.0011
	5	*k*: 0.687, *n*: 0.687	0.997	0.0141	0.0002
Henderson	1	*k*: 0.005, *a*: 0.935	0.982	0.0414	0.002
And Pabis	3	*k*: 0.016, *a*: 0.903	0.878	0.0921	0.0102
	5	*k*: 0.054, *a*: 0.995	0.963	0.0547	0.0037
Midilli et al.	1	*k*: −10.459, *a*: 0.00005, *n*: −0.024, *b*: 0.000	0.981	0.0338	0.0016
	3	*k*: 0.420, *a*: 1.000, *n*: 0.243, *b*: 0.000	0.999	0.0091	0.0001
	5	*k*: 1.207, *a*: 1.000, *n*: 0.125, *b*: 0.000	1.000	0	0

The Page model however provided a good agreement between the experimental and the predicted data sets for the blanched samples. The correlation coefficients obtained for the three models are in the range of 0.955–0.994. Unlike the microwave pretreated samples, the values of the drying rate constant, *k,* decreased with the increase in blanching time, indicating a reduction in drying rate potential with blanching duration. The estimated parameters and statistical analysis of the models examined for the different drying conditions are illustrated in Table 5.

**Table 5:** Parameters and statistical results for the various drying models for blanched samples dried at 70°C

Model name	*P*_t_	Model constants	*R* ^2^	RMSE	*χ* ^2^
Page	1	k: 0.000, n: 1.419	0.983	0.0447	0.0023
	3	k: 0.000, n: 1.442	0.980	0.0480	0.0027
	5	k: 0.000, n: 1.565	0.994	0.0270	0.0008
Henderson	1	k: 0.004 a: 1.044	0.962	0.0681	0.0054
And Pabis	3	*k: 0.003 a*: 1.043* *	0.955	0.0712	0.0060
	5	k: 0.003 a: 1.079	0.962	0.0683	0.0053
Midilli et al.	1	*k: −8.644,* a: 0.000*, n: −0.019, b: 0.000*	0.967	0.0554	0.0043
	3	k: 11.899, a: 0.905, n: −34.503, b: −0.002	0.981	0.0411	0.0024
	5	k: 12.562, a: 0.905, n: −36.508, b: −0.001	0.955	0.0673	0.0061

### Influence of different pretreatments and air temperature on drying time

**Figure 5:**
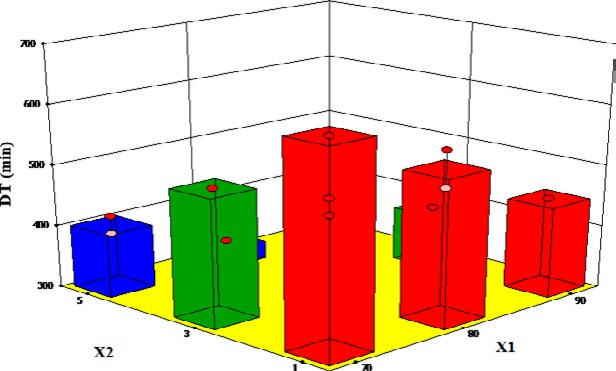
Effect of temperature and microwave pretreatment time on drying time.

**Figure 6:**
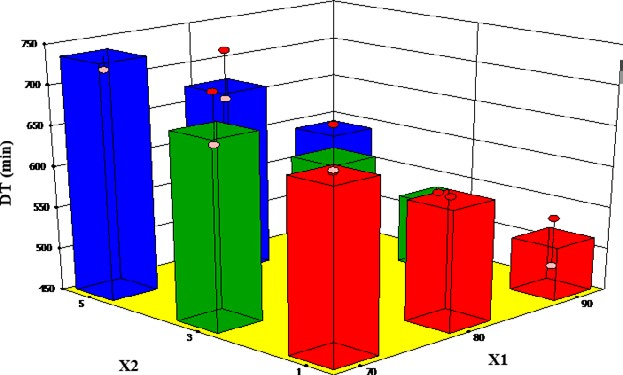
Effect of air temperature and blanching time on drying time.

The effect of air temperature, microwave time, and blanching time are significant model terms on the drying time of yam cubes (Tables 6 and 7). It can be observed from Figure 5 that as air temperature and microwave pretreatment time increased, drying time also decreased. Drying time was lowest at air temperature of 90°C and microwave pretreatment time of 5 min. These results agree with drying of potato slices by Akpinar et al. (2003) and apple pomace by Motevali et al. (2010). Longer blanching times however increased the drying time (Fig. 6).

**Table 6:** Analysis of variance (ANOVA) for the effects of microwave time and hot air temperature on drying time

Source	Coefficient estimates	SS	df	Mean square	*F*‐value	*P*‐value, prob > *F*
Intercept	448.33	**–**	**–**	**–**	**–**	**–**
Model	–	142,200	8	17,771.69	63.19	<0.0001^a^
*X*_1_	66.67	47,134.62	2	23,567.31	83.79	<0.0001^a^
*X*_2_	91.67	88,564.62	2	44,282.31	157.45	<0.0001^a^
*X*_1_ *X*_2_	23.33	6415.38	4	1603.85	5.70	0.0180^a^
*R* ^2^	0.9844					

SS, sum of squares; df, degree of freedom.

a Significant (<0.0500).

**Table 7:** Analysis of variance (ANOVA) for the effects of blanching time and hot air temperature on drying time

Source	Coefficient estimates	SS	df	Mean square	*F*‐value	*P*‐value, prob > *F*
Intercept	623.08	–	–	–	–	
Model	–	79,438.91	4	19,859.73	32.27	<0.0001^a^
*X*_1_	66.92	53,335.38	2	26,667.69	43.34	<0.0001^a^
*X*_2_	−33.08	17,815.38	2	8907.69	14.47	<0.0006^a^
*R* ^2^	0.9149					

SS, sum of squares; df, degree of freedom.

a Significant (<0.0500). Lack of fit is insignificant.

### Influence of different pretreatments and air temperature on nonenzymatic browning

**Figure 7:**
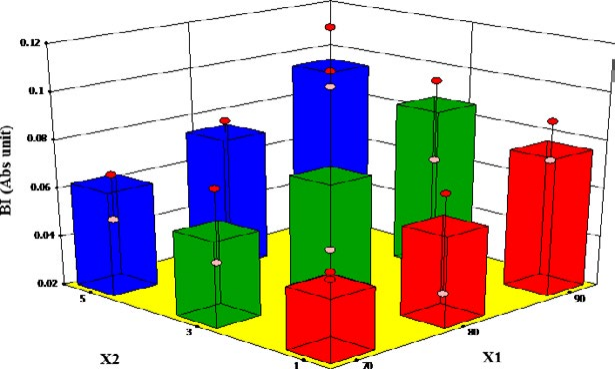
Effect of temperature and pretreatment time (microwave) on browning index (BI).

Browning in yam is caused by both enzymatic and nonenzymatic reactions. However, the former was suppressed with the microwave and blanching pretreatments. Therefore, brown pigment formation in the dried yam was assumed to be due to nonenzymatic browning. The nonenzymatic browning index (BI) is a quality indicator for browning in white yam during drying. The extent of browning is mainly attributed to the color changes resulting from reactions between reducing sugars and nitrogenous compounds in the yam (Cernîsev 2010). The influence of microwave pretreatment time and hot air drying temperatures on the development of nonenzymatic browning in white yam was evident (Fig. 7). Browning index increased with both microwave pretreatment time and temperature from 0.012 in the fresh yams to 0.112 after drying at 90°C and microwave pretreatment time of 5 min. This increasing BI with both microwave pretreatment time and temperature indicates that the yam cubes were greatly affected by microwave pretreatment time and hot air temperature. The brown pigment formation in the dried yam cubes is due to the reactions between nitrogenous constituents and reducing sugars, nitrogenous constituents and organic acids, and sugars and organic acids (Zanoni et al. 1999).

**Figure 8:**
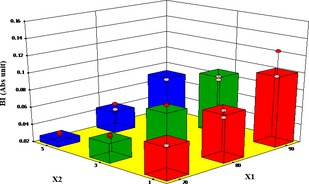
Effect of temperature and blanching time on browning index  BI).

However, in the case of the blanched samples, BI decreased with increase in pretreatment time from 0.127 at 90°C and blanching time of 1 min to 0.029 after drying at 70°C and blanching time of 5 min (Fig. 8). Namtip et al. (2006) blanched and dried sweet potato chips at 70°C–90°C and observed that the rate of browning increased with temperature from 0.039 to 0.755. Moreover, the response of the non‐enzymatic browning index (BI) model and the ANOVA results displayed in Table 8 clearly show that air temperature and microwave time are insignificant model terms for the microwave dried yam cubes. However, in the case of the blanched samples, air temperature and blanching time are significant model term on the nonenzymatic browning of yam cubes during drying (Table 9).

**Table 8:** Analysis of variance (ANOVA) for the effects of microwave time and hot air temperature on nonenzymatic browning

Source	Coefficient estimates	SS	df	Mean square	*F*‐value	*P*‐value, prob > *F*
Intercept	0.069	–	–	–	–	
Model	–	0.004086	4	0.001021	2.14	0.1376^a^
*X*_1_	−0.015	0.003136	2	0.001568	3.29	0.0724^a^
*X*_2_	−0.009231	0.0008837	2	0.0004418	0.93	0.4220^a^
*R* ^2^	0.9169					

SS, sum of squares; df, degree of freedom.

a Significant (<0.0500). Lack of fit is not significant.

**Table 9:** Analysis of variance (ANOVA) for the effects of blanching time and hot air temperature on nonenzymatic browning

Source	Coefficient estimates	SS	df	Mean square	*F*‐value	*P*‐value, prob > *F*
Intercept	0.064					
Model		0.008855	4	0.002214	27.67	<0.0001^a^
*X*_1_	−0.021	0.005597	2	0.002798	34.98	<0.0001^a^
*X*_2_	0.015	0.002371	2	0.0001185	14.82	<0.0006^a^
*R* ^2^	0.9022					

SS, sum of squares; df, degree of freedom.

a Significant (<0.0500). Lack of fit is not significant.

### Influence of drying conditions on ascorbic acid content

**Figure 9:**
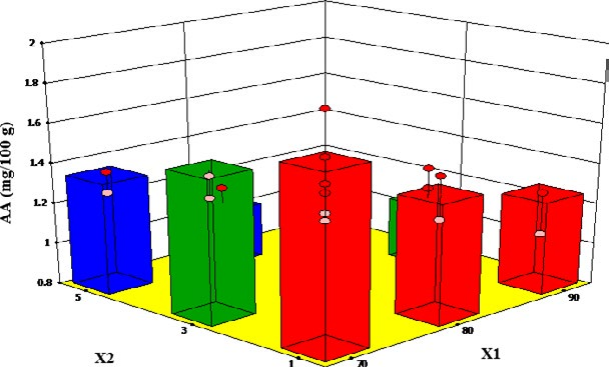
Effect of temperature and pretreatment time (microwave) on ascorbic acid.

**Figure 10:**
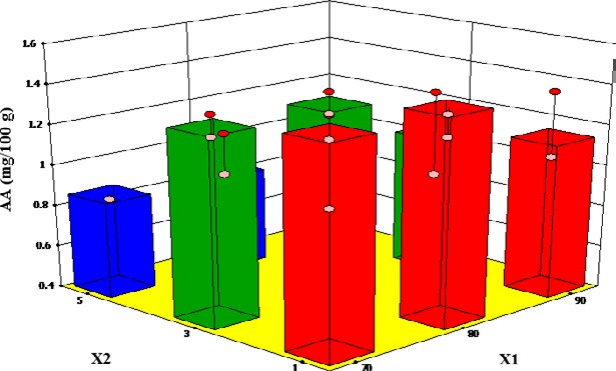
Effect of temperature and pretreatment time (blanching) on ascorbic acid content.

The effect of microwave pretreatment time (MT) and blanching time (BT) at the various air temperatures on ascorbic acid (AA) of white yam are illustrated in Figures 9 and 10, respectively. Both MT and BT were significant model terms on the ascorbic acid (AA) content (Tables 10 and 11). Longer MT and BT resulted in more reduction of the AA content. The AA content of the fresh yam decreased from 4.33 mg/100 g dry matter to 0.528 mg/100 g dry matter after blanching for 5 min and drying at 90°C representing 88% loss in vitamin C. The reduction in ascorbic acid content observed for the microwave pretreated samples may be due to the destruction of vitamin C by the electromagnetic waves. The thermal effect of the microwave power coupled with the irreversible oxidative reactions due to longer drying times during hot air drying may have contributed to the excessive damage of the AA content.

**Table 10:** Analysis of variance (ANOVA) for the effects of microwave time and hot air temperature on ascorbic acid content

Source	Coefficient estimates	SS	df	Mean square	*F*‐value	*P*‐value, prob > *F*
Intercept	1.29	–	–	–	–	
Model	–	0.76	4	0.19	10.73	0.0006^a^
*X*_1_	0.23	0.51	2	0.26	14.38	0.0007^a^
*X*_2_	0.16	0.26	2	0.13	7.44	0.0079^a^
*R* ^2^	0.7815	–		–	–	–

SS, sum of squares; df, degree of freedom.

a Significant (<0.0500) Lack of fit was not significant.

**Table 11:** Analysis of variance (ANOVA) for the effects of blanching time and hot air temperature on Ascorbic acid content

Source	Coefficient estimates	SS	df	Mean square	*F*‐value	*P*‐value, prob > *F*
Intercept	1.11	–	–	–	–	
Model	–	0.96	4	0.24	12.54	0.0003^a^
*X*_1_	0.083	0.21	2	0.11	5.57	0.0194^a^
*X*_2_	0.21	0.84	2	0.42	21.99	*<*0.0001^a^
*R* ^2^	0.8070	–		–	–	–

SS, sum of squares; df, degree of freedom.

a Significant (<0.0500) lack of fit was not significant.

Similarly, there were decreases in the AA content in the blanched samples with blanching time and temperature. This may be due to its high solubility and susceptibility to heat. Losses of ascorbic acid during microwaving and blanching have been reported by Oladele and Aborisade (2009), Marfil et al. (2008) and Rickman et al. (2007). The reduction agrees with results obtained by (Zheng and Lu 2011) for microwave pretreatment on the AA in different parts of green asparagus. According to Sokhansanj and Jayas (1995), the loss of AA content during drying of food stuffs was between 10% and 50%. Daood et al. (1996) found 63% reduction in AA during the drying of red peppers at ambient temperatures. In a related study involving microwave drying of okra fruit. Mana et al. (2012) reported AA reduction between 43% and 63%. Leng et al. (2011) reported more than 50% AA losses after blanching and drying of *Dioscorea schimperina*. Ascorbic acid Losses as high as 92.29% and 96.29% were realized for blanching *Solanum gilo* and *Gnetum africanum*, respectively (Enemo et al. 2010).

### Optimization of the drying parameters

The optimal microwave and blanching drying conditions were established using the concept of the overall desirability index in equation (8). The maximum predicted DT, BI, and AA were 432.35 min, 0.0893 Abs unit, and 1.5157 mg/100 g, respectively, for the microwave drying conditions. These simulated values are closer to their corresponding experimental values of 630 min DT, 0.112 BI, and 1.9008 mg/100 g dry matter AA content. However, for the blanched samples, the maximum predicted DT, BI, and AA were 625.63 min, 0.0854 Abs unit, and 1.4026 mg/100 g, respectively. These simulated values are closer to their corresponding experimental values of 720 min DT, 0.127 BI, and 1.4784 mg/100 g dry matter AA content.

**Figure 11:**
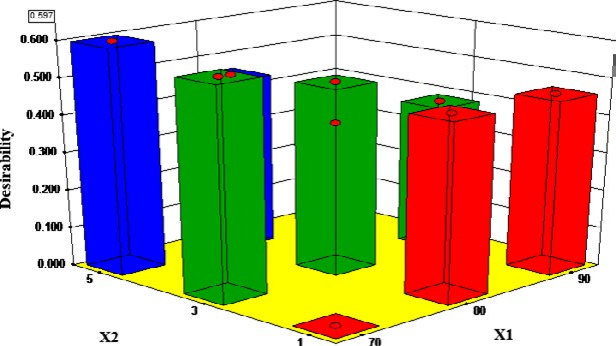
Effect of air temperature and microwave pretreatment time on 3‐D plot of the desirability index for the optimal drying condition.

**Figure 12:**
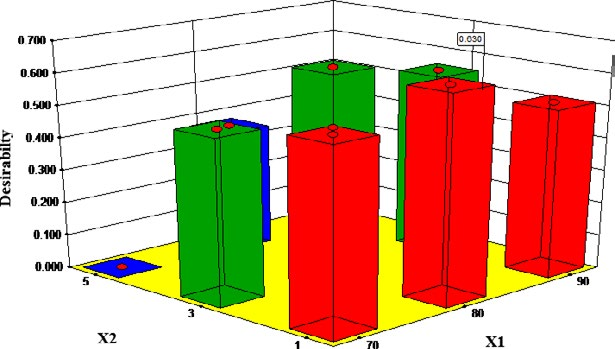
Effect of air temperature and blanching time on 3‐D plot of the desirability index for the optimal drying condition.

The overall desirability of 0.60 and 0.63 was obtained for the respective microwave and blanching effect on the quality of dried samples (Figs. 11 and 12). The results predicted with 95% confidence in the range of the independent variables gave optimal temperature of 70°C and MT of 5 min for microwave‐assisted drying and a temperature of 80°C and BT of 1 min for the blanched‐assisted drying. At this optimum condition, the DT was 405 min, the BI 0.0635 Abs unit, and the AA was 1.358 mg/100 g dry matter, while a DT of 596 min, BI of 0.0750 Abs unit, and AA of 1.403 mg/100 g dry matter were obtained for the blanched samples.

## Conclusion

It can be concluded that both microwave and blanching time had a profound effect on the drying time, nonenzymatic browning and ascorbic acid content of white yam during drying. The drying time reduced from 630 to 330 min as the microwave time increased from 1 to 5 min but blanching increased the drying time from 480 to 720 min. The microwave pretreatment led to higher browning and lesser reduction in ascorbic acid content of the yam cubes blanching. The moisture diffusivity coefficient increased with increasing microwave time and hot air temperature but decreased with increasing blanching time. Among the three thin‐layer drying models that were fitted to the experimental data, the Midilli et al. model showed the best fit for the microwave‐assisted drying of yam cubes while the Page model gave the best fit for the blanched samples. The study shows that microwave drying can be used to enhance drying potential and produce better quality dried yam for food preparation and pharmaceutical formulations. The optimized results provide options for industrial drying of yam.

## Conflict of Interest

None declared.
